# Supplementary material for: D-DI/PLT can be a prognostic indicator for sepsis
Source: PeerJ. 2023 Sep 5;11:e15910. doi: 10.7717/peerj.15910 (PMC10487589; doi:10.7717/peerj.15910)
Supplement: Supplemental Information 1 [file peerj-11-15910-s001.docx]

## Supplementary Tables:

**Supplementary Table 1. Disease-related clinical indicators of sepsis (*p*>0.05)**

| **Indicators** | Survival (n=141) | Dead (n=60) | *P* |
| --- | --- | --- | --- |
| **Inflammatory** |  |  |  |
| NEU (×10^9^/L) | 10.42 ± 5.87 | 12.55 ± 8.46 | 0.08 |
| LYM (×10^9^/L) | 0.95 (0.71, 1.54) | 0.97 (0.60, 1.43) | 0.40 |
| RDW (fl) | 46.89 ± 7.53 | 48.89 ± 8.53 | 0.12 |
| IL-6 (pg/mL) | 128.81 ± 189.72 | 118.68 ± 172.30 | 0.71 |
| PCT (ng/mL) | 0.47 (0.14, 4.19) | 0.59 (0.22, 2.71) | 0.97 |
| **Coagulation** |  |  |  |
| APTT (s) | 16.56±3.05 | 17.55 ± 3.24 | 0.05 |
| FIB (G/L) | 3.76 ± 2.25 | 3.47 ± 2.50 | 0.44 |
| **Ratio** |  |  |  |
| RLR | 47.01 (31.11, 65.26) | 51.92 (33.76, 76.52) | 0.16 |
| RDW/ALB | 1.50 ± 0.43 | 1.59 ± 0.46 | 0.19 |
| **Pulmonary Function** |  |  |  |
| PCO_2_ (mmHg) | 31.82 ± 12.51 | 30.62 ± 13.97 | 0.56 |
| CO_2_ (mmHg) | 22.13 ± 5.86 | 23.8 ± 5.29 | 0.05 |
| pH | 7.43 (7.38,7.49) | 7.45 (7.35,7.50) | 0.82 |
| **Circulation Function** |  |  |  |
| CnT-I (ng/mL) | 0.05 (0.02, 0.34) | 0.07 (0.03, 0.86) | 0.07 |
| MB (ng/mL) | 1140.72 ± 10301.91 | 495.08 ± 1350.71 | 0.47 |
| LDH (IU/L) | 447.35 ± 574.04 | 480.20 ± 491.03 | 0.68 |
| **Liver Function** |  |  |  |
| TBIL (umol/L) | 31.76 ± ±54.96 | 35.67 ± 68.22 | 0.70 |
| ALB (g/L) | 32.77 ± 6.98 | 32.59 ± 8.44 | 0.89 |
| AST (IU/L) | 87.86 ± 271.70 | 119.38 ± 319.20 | 0.51 |
| ALT (IU/L) | 108.51 ± 338.77 | 77.47 ± 121.01 | 0.34 |

Notes: NEU= Absolute Neutrophil Count; LYM= Absolute Lymphocyte Count; RDW= Red Cell Distribution Width; IL-6= Interleukin 6; PCT= Procalcitonin; APTT= Activated Partial Thromboplastin Time; FIB= Fibrinogen; RLR= Red Blood Cell Distribution Width to Lymphocyte Ratio; RDW/ALB= Red Blood Cell Distribution Width to Albumin Ratio; PCO_2_= Partial Pressure of Carbon Dioxide; CO_2_= Blood Concentration of Carbon Dioxide; pH= potential of Hydrogen; CnT-I= Cardiac Troponin I; MB= Myoglobin; LDH= Lactate Dehydrogenase; TBIL= Total Bilirubin; ALB= Albumin; ALT= Glutamic-pyruvic Transaminase; AST= Glutamic Oxalacetic Transaminase.

**Supplementary Table 2. Result of Cox stepwise regression**

| Tag | Hazard Ratio | lower 95%CI | upper 95%CI | *P*-value |
| --- | --- | --- | --- | --- |
| NEWS | 0.91 | 0.81 | 1.01 | 0.07 |
| RPR | 4.02 | 0.79 | 20.44 | 0.09 |
| UA | 1.00 | 1.00 | 1.00 | 0.12 |
| PLR | 1.00 | 1.00 | 1.01 | 0.14 |
| PO₂ | 1.01 | 1.00 | 1.02 | 0.16 |
| NLR | 0.98 | 0.94 | 1.01 | 0.16 |
| Urea | 0.98 | 0.95 | 1.02 | 0.28 |
| PT | 1.06 | 0.95 | 1.20 | 0.29 |
| SBP | 0.99 | 0.98 | 1.01 | 0.36 |
| DBP | 1.01 | 0.99 | 1.04 | 0.42 |
| BNP | 1.00 | 1.00 | 1.00 | 0.52 |
| Ccr | 1.00 | 1.00 | 1.00 | 0.59 |
| CK-MB | 1.00 | 0.99 | 1.01 | 0.84 |
| Lac | 1.00 | 0.89 | 1.12 | 0.97 |
