# Supplementary material for: D-DI/PLT can be a prognostic indicator for sepsis
Source: PeerJ. 2023 Sep 5;11:e15910. doi: 10.7717/peerj.15910 (PMC10487589; doi:10.7717/peerj.15910)
Supplement: Supplemental Information 2 — (A) Spearman correlation analysis of the association between D-DI/PLT and age. R represents the correlation coefficient. (B) The D-DI/PLT ratio was compared between male and female patients with sepsis. (C) D-DI/PLT was compared between sepsis patients with and without hypertension. (D) D-DI/PLT was compared between sepsis patients with and without diabetes. (E) Comparison of D-DI/PLT in different groups of patients with sepsis according to grouping of pathogenic infection types. The data with non-normal distribution are represented by box plots. The P value of less than 0.05 indicates statistical significance. [file peerj-11-15910-s002.pdf]

Supplementary Material

Supplementary Figure

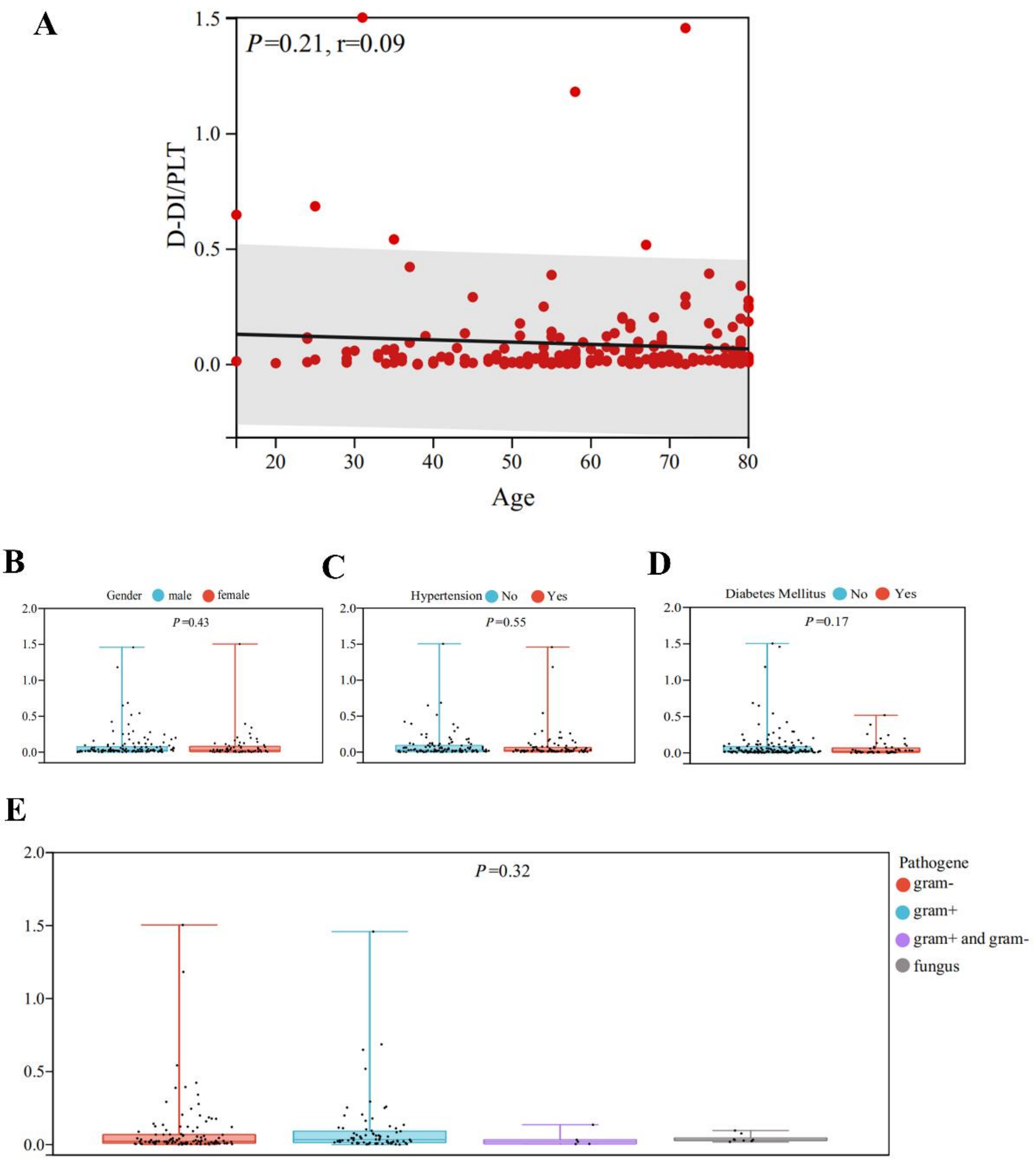

Fig.S1

Supplementary Figure 1. Comparison of D-DI/PLT in the analysis of different subgroups. (A), Spearman correlation analysis of the association between D-DI/PLT and age. R represents the correlation coefficient. (B), The D-DI/PLT ratio was compared between male and female patients with sepsis. (C) D-DI/PLT was compared between sepsis patients with and without hypertension. (D) D-DI/PLT was compared between sepsis patients with and without diabetes. (E) Comparison of D-DI/PLT in different groups of patients with sepsis according to grouping of pathogenic infection types. The data with non-normal distribution are represented by box plots. The P value of less than 0.05 indicates statistical significance.
