# Supplementary material for: D-DI/PLT can be a prognostic indicator for sepsis
Source: PeerJ. 2023 Sep 5;11:e15910. doi: 10.7717/peerj.15910 (PMC10487589; doi:10.7717/peerj.15910)
Supplement: Supplemental Information 4 [file peerj-11-15910-s004.docx]

**The meaning of the number represented by the categorical data**

In the outcome category, 0 represents patients who are alive and 1 represents patients who are dead

In the gender classification, 1 represents male and 0 represents female

Among the underlying diseases, 1 was defined as having diabetes and 0 was defined as not having diabetes

Whether hypertension was present in the underlying disease, 1 means yes and 0 means no

**Survival curves were grouped according to the criteria**

A **DDI/PLT** ratio of 0.07 was used as the cutoff value for grouping, with 1 indicating a DDI/PLT less than 0.07 and 2 indicating a value greater than or equal to 0.07

A **DDI** value of 5.54 was used as the cutoff value for grouping, with 1 indicating a DDI/PLT less than 5.54 and 2 indicating a value greater than or equal to 5.54

A **SOFA** value of 11.5 was used as the cutoff value for grouping, with 1 indicating that the SOFA total score was less than 11.5 and 2 indicating that the value was greater than or equal to 11.5

A **PLT** count value of 161.5 was used as the cutoff value for grouping, with 1 indicating less than 161.5 and 2 indicating a value greater than or equal to 161.5
